# Studying Müllerian duct anomalies – from cataloguing phenotypes to discovering causation

**DOI:** 10.1242/dmm.047977

**Published:** 2021-06-23

**Authors:** Laura Santana González, Mara Artibani, Ahmed Ashour Ahmed

**Affiliations:** 1 MRC Weatherall Institute of Molecular Medicine, University of Oxford, Oxford OX3 9DS, UK; 2 Nuffield Department of Women's and Reproductive Health, University of Oxford, Oxford OX3 9DU, UK; 3 Gene Regulatory Networks in Development and Disease Laboratory, MRC Weatherall Institute of Molecular Medicine, Radcliffe Department of Medicine, University of Oxford, Oxford OX3 9DS, UK

**Keywords:** Developmental model systems, Heterogeneous disorders, Müllerian development, Müllerian duct anomalies, State-of-the-art technologies

## Abstract

Müllerian duct anomalies (MDAs) are developmental disorders of the Müllerian duct, the embryonic anlage of most of the female reproductive tract. The prevalence of MDAs is 6.7% in the general female population and 16.7% in women who exhibit recurrent miscarriages. Individuals affected by these anomalies suffer from high rates of infertility, first-trimester pregnancy losses, premature labour, placental retention, foetal growth retardation and foetal malpresentations. The aetiology of MDAs is complex and heterogeneous, displaying a range of clinical pictures that generally lack a direct genotype-phenotype correlation. *De novo* and familial cases sharing the same genomic lesions have been reported. The familial cases follow an autosomal-dominant inheritance, with reduced penetrance and variable expressivity. Furthermore, few genetic factors and molecular pathways underpinning Müllerian development and dysregulations causing MDAs have been identified. The current knowledge in this field predominantly derives from loss-of-function experiments in mouse and chicken models, as well as from human genetic association studies using traditional approaches, such as microarrays and Sanger sequencing, limiting the discovery of causal factors to few genetic entities from the coding genome. In this Review, we summarise the current state of the field, discuss limitations in the number of studies and patient samples that have stalled progress, and review how the development of new technologies provides a unique opportunity to overcome these limitations. Furthermore, we discuss how these new technologies can improve functional validation of potential causative alterations in MDAs.

## Abstract (precis)

**Summary:** Here, we review the current knowledge about Müllerian duct anomalies in the context of new high-throughput technologies and model systems and their implications in the prevention of these disorders.

## Introduction

Müllerian duct anomalies (MDAs) represent a range of congenital aberrations of the female reproductive tract that result in many pathologies in obstetrics and gynaecology, such as infertility, recurrent first-semester miscarriage, foetal malpresentations and intrauterine growth restriction, as well as premature delivery, placental retention and increased need for caesarean section delivery (Rock and Schlaff, 1985; Lin et al., 2002; Green and Harris, 1976; Maneschi et al., 1995; Acién, 1993). The prevalence of MDAs among the normal female population is ∼6.7%, although some populational clusters differ. For example, infertile females show an MDA prevalence of 7.3%, and women exhibiting recurrent spontaneous miscarriages show a prevalence of 16.7% (Saravelos et al., 2008). MDAs are developmental disorders emerging from defects in organogenesis and subsequent differentiation of the Müllerian ducts, also called the paramesonephric ducts, which are the embryonic anlage of the majority of the female reproductive tract: fallopian tubes or oviducts, uterus or uterine horns, and the upper two-thirds of the vagina. Despite their clinical significance, the aetiology (see Glossary, Box 1) and pathogenesis of MDAs are poorly understood, partly due to their complexity and partly due to limitations in the number of studies and patient samples. MDAs exhibit a heterogeneous spectrum of clinical presentations ranging from isolated defects of a single reproductive organ to multiorgan aplasia (Box 1). These can occur in combination with other system aberrations or other syndromes, which will be presented in detail in this Review (Grimbizis et al., 2013; Strübbe et al., 1992; Duncan et al., 2020; Morcel et al., 2007; Fontana et al., 2017; Guerrier et al., 2006; Herlin et al., 2020). An added complication is the variable phenotypic penetrance of MDAs, leading to inconsistent relationships between genotype and phenotype, whereby the same genomic aberration results in a broad spectrum of clinical pictures. This suggests that the aetiology of these anomalies is driven by additional factors beyond genetic determinants.

This work reviews Müllerian development mechanisms, the derived human anomalies and syndromes, their characteristics and causative components. Furthermore, this Review assesses the latest progress in this field and the new opportunities that can translate into substantial improvement in investigating MDA aetiology, diagnosis, treatment and prevention, including genetic counselling. Moreover, we discuss how, despite the putative multifactorial components and challenges surrounding these heterogeneous anomalies, the field achieved recent progress. Several genomic copy number variations (CNVs; Box 1) and causative genetic variants have been identified, fuelled by the recent progress in the discovery of gene regulatory networks (GRNs; Box 1) and molecular pathways underpinning Müllerian development and differentiation in model systems such as murine and chicken embryos. We further summarise how human genetic association studies using microarrays, fluorescence *in situ* hybridisation (FISH) and Sanger sequencing (Box 1) have identified many genes associated with MDAs. The dominance of low-throughput techniques has limited discoveries in this field, and the current genomics revolution provides a unique opportunity to implement state-of-the-art high-throughput next-generation sequencing (NGS; Box 1) approaches to overcome these limitations. However, as many of the traditional low-throughput techniques remain highly important for validation of causation, an integrated approach would be optimal. Furthermore, functional validation of potential causative alterations in MDAs is now easier due to the development of new experimental model systems and genetic toolkits.

**Box 1. Glossary**

**3D organoid model:** a stem cell-derived 3D *in vitro* model faithfully mimicking the organ of origin. It contains a variable number of cells and, depending on complexity, some or all cell types found in the organ of origin.

**Aetiology:** the cause or causes of a given disease or pathological condition.

**Agenesis:** failure of an organ to develop during embryonic growth and development.

**Al-Awadi/Raas-Rothschild/Schinzel phocomelia syndrome (AARR; OMIM 276820):** a rare developmental disorder characterised by limb/pelvic abnormal development or agenesis. Furthermore, patients with AARR present with renal and genital anomalies and facial malformations.

**Aplasia:** failure of an organ or tissue to develop or to function normally.

**Atresia:** a condition in which an orifice or passage in the body is closed or absent.

**Autosomal dominance:** inheritance pattern of some genetic disorders. The affected gene is in an autosomal/non-sex chromosome (chromosomes 1-22 in humans) and a single copy of this mutant gene is enough to cause the disease. In recessive disorders, both alleles need to be mutated to cause the disorder.

**Bone morphogenetic protein (BMP) signalling pathway:** pathway triggered by the binding of a BMP molecule to a receptor on the target cell. It is involved in numerous developmental processes.

**Copy number variations (CNVs):** sections of the genome presenting at different copy numbers compared to the reference genome due to gains or losses of genomic material.

**Dach genes:** the Dach/dachshund family of transcriptional cofactors is conserved in insects and vertebrates. These genes are expressed in several organs during embryonic development.

**DiGeorge syndrome (22q11.2 deletion syndrome; OMIM 188400):** a 3-4 Mb deletion that phenotypically presents a complex clinicopathological picture, with heart disorders, hypocalcaemia, immunodeficiency, and cognitive and behavioural difficulties.

**Dmrt1:** a conserved transcription factor containing a zinc finger-like DNA-binding domain (DM domain). It is expressed in the Müllerian mesenchymal lineages.

**DNase1-hypersensitive sites:** chromatin regions that are less compacted and are accessible to proteins such as DNases and transcription factors. They indicate sites of active DNA transcription.

**Duplex PCR/liquid chromatography (DP/LC):** method used to detect CNVs. It uses multiplex PCR to amplify the required exons, followed by liquid chromatography to separate and quantify them.

**Emx2:** a transcription factor containing a homeobox sequence. It is mainly expressed in the dorsal telencephalon, olfactory neuroepithelium and urogenital system. It plays key roles in several developmental mechanisms.

**Epithelial-mesenchymal transition (EMT):** a mechanism by which a polarised epithelial cell on a basal membrane can convert into a non-polarised mesenchymal cell, becoming more resistant to apoptosis, prone to migration and developing further mesenchymal features.

**Fibroblast growth factor (FGF) pathway:** a signalling pathway activated by the union of any of the 22 FGF ligands to their FGF receptors (FGFRs). This pathway governs a range of cellular processes such as proliferation, differentiation and embryonic development.

**Fluorescence *in situ* hybridisation (FISH):** a molecular technique in which fluorescent nucleotide probes bind to complementary nucleic acid sequences in a cell or tissue. It is often used to detect gains, losses and chromosomal arm translocations in karyotypes.

**Gata3:** a transcription factor belonging to the GATA family. It contains two zinc finger regions of the GATA type and has been reported to act in T-cell, breast and endothelial differentiation.

**Gene regulatory networks (GRNs):** complex of molecular regulators that control the expression of certain genetic identities that are key for a given biological process.

**Gpr56:** a G protein-coupled receptor and member of the adhesion GPCR family.

**Hnf1b:** a POU homeodomain transcription factor strongly expressed in the mesoepithelial lineage during murine Müllerian duct development and in the adult differentiated oviducts and uterine horns.

**Hox genes:** members of the homeobox family of conserved embryonic transcription factors that specify regions of the embryonic body along the proximal-caudal axis.

***KLHL4*:** a recently discovered gene in humans belonging to the kelch family. It contains kelch-repeat motives, which interact with actin, as well as a POZ/BTB protein-binding domain.

**Leydig cell:** male cells of the testicles located next to the seminiferous tubules. They are in charge of producing and secreting androgens (testosterone).

**Lim1:** a LIM homeobox transcription factor with one homeobox domain and two amino-terminal cysteine-histidine-containing LIM domains that bind iron and zinc.

**Maturity-onset diabetes of the young, type 5 [MODY5; also known as renal cyst and diabetes syndrome (RCAD); OMIM 137920]:** a rare disease characterised by mild diabetes and renal and genital malformations.

**Mesonephric coelomic epithelium:** the epithelial layer surrounding the mesonephros. It contains the progenitor cells to the Müllerian ducts' components and the ovarian epithelium.

**Mesonephros:** the middle of the three kidneys (pronephros, mesonephros and metanephros) that sequentially arise in the embryo during development. It only functions during embryonic development in Aves and mammals, where the metanephros is the adult renal organ that differentiates into the kidney.

**Microarrays:** genetic tool in which nucleic acids from a reference organism are arrayed on a solid surface for hybridisation with a sample containing target genomic DNA or RNA.

**Misr2:** a receptor for the anti-Müllerian hormone (AMH). This hormone and testosterone are required for sexual male differentiation. The binding of AMH to Misr2 triggers Müllerian duct regression in male embryos.

**Multiplex ligation-dependent probe amplification (MPLA):** method based on the use of multiple probes targeting regions of the genome. It is used to detect genomic aberrations.

**Next-generation sequencing (NGS):** a term describing a combination of modern high-throughput DNA and RNA sequencing techniques.

**Pax2/Pax8:** members of the PAX family of transcription factors. They share a high degree of sequence similarity and contain a paired box and octapeptide domain as well as an incomplete homeodomain missing the DNA-binding section.

**Pbx1:** a PBX homeobox transcription factor. Suggested to play a role in osteogenesis and skeletal patterning additionally to urogenital system development.

**Phosphoinositide 3-kinase (PI3K) pathway:** an important conserved pathway that regulates cell metabolism, proliferation, survival and growth.

***RBM8A*:** a gene encoding Y14, which has a conserved RNA-binding motif and is part of the exon-junction complex.

**Reduced penetrance and variable expressivity:** reduced or incomplete penetrance refers to the fact that some patients do not develop symptoms/features of the genetic disease even though they carry the mutated allele. Variable expressivity indicates that the symptoms of the same genetic damage are very heterogeneous and change among different patients. Reduced penetrance and variable expressivity occur due to external non-genetic variables.

**Sanger sequencing:** low-scale DNA-sequencing method based on fluorescent chain-terminating nucleotides during replication of the target DNA. This produces a range of DNA fragments that can be read and allow the interrogated sequence to be determined.

**Thrombocytopenia-absent radius syndrome (TAR; OMIM 27400):** a rare developmental disorder characterised by a low level of platelets in the blood and agenesis of the radius.

**TBX6:** the transcription factor TBX6 is a developmental master regulator with a conserved DNA-binding T-box domain.

**Whole-genome sequencing (WGS)/whole-exome sequencing (WES):** high-throughput DNA sequencing methods. WGS reads the complete genome of an individual, including mitochondrial DNA, whereas WES only reads the exons, so the coding regions, of the genome.

**Wnt family:** family of 19 secreted glycoproteins that display a range of conserved functions, from embryonic processes to cell differentiation and systemic tissue homeostasis.

**Wolffian or mesonephric ducts:** primordial male genital ducts that develop into the male reproductive system (epididymis, vas deferens, seminal vesicle and ejaculatory duct).

## Müllerian duct development: genetics, molecular and cellular mechanisms

Müllerian organogenesis takes place in the mesonephros (Box 1), a primitive part of the embryonic urogenital system that also hosts the Wolffian ducts (Box 1), the early male reproductive tubes. Both Müllerian and Wolffian ducts are temporary primordial reproductive sets of tubes that arise during embryonic development. The structures that correspond to the genetic sex of the embryo are conserved and develop into a functional reproductive tract, whereas the sex-unmatching tubes regress (Dohr and Tarmann, 1984; Jacob et al., 1999; Gruenwald, 1941; Guioli et al., 2007; Orvis and Behringer, 2007; Atsuta and Takahashi, 2016). Hence, the Müllerian ducts develop and remain in female embryos.

**Fig. 1.**
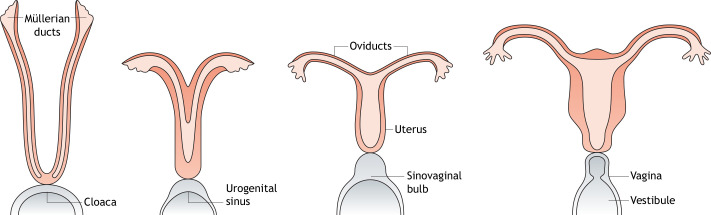
**Embryonic formation of the Müllerian ducts in humans.** The Müllerian ducts originate during embryonic development as two separate tubes that undergo midline fusion, septal resorption and eventually fuse with the urogenital sinus. This embryonic structure finally differentiates into the female reproductive tract.

After their organogenesis in humans, both tubes undergo midline fusion, septal resorption and eventually fuse with the urogenital sinus (Fig. 1). The complete process is executed in three chronological stages between developmental weeks 7 and 8 (Carnegie stage 18-23) (Hashimoto, 2003). Although, this has been reviewed in detail elsewhere (Mullen and Behringer, 2014; Cunha et al., 2018; Roly et al., 2018; Santana González et al., 2021), we provide a brief summary here. First, specific progenitors on the mesonephric coelomic epithelium (Box 1) commit to a Müllerian fate (stage I) and subsequently invaginate into the mesonephric mesenchyme (stage II). Müllerian genesis is then completed by a caudal elongation of the tubes along the anteroposterior mesonephric axis, between the Wolffian duct and the mesonephric coelomic epithelial sheet (stage III). Although every developmental phase occurs in proximity to the Wolffian ducts, only stage III demonstrably relies on Wolffian signalling (Kobayashi et al., 2005).

The earliest sign of Müllerian development is epithelial thickening of some coelomic epithelial cells, called the Müllerian ridge, which contains epithelial and mesenchymal lineage progenitors, the formation of which depends on the bone morphogenetic protein (BMP) signalling pathway (Box 1) (Atsuta and Takahashi, 2016). The Müllerian ridge is the only niche for the epithelial progenitors (Ghosh et al., 2020) that give rise to the epithelium lining the inside of the tubes and forming the lumen. However, the mesenchymal lineages of the Müllerian stroma derive from progenitors in the Müllerian ridge and progenitors located along the length of the mesonephric coelomic epithelium (Guioli et al., 2007; Ayers et al., 2015; Prunskaite-Hyyryläinen et al., 2016). Müllerian progenitors are marked by the expression of embryonic transcription factors such as Pax2, Pax8, Emx2, Lim1, Pbx1 and Hnf1b (Box 1) in epithelial lineage progenitors and Dmrt1 (Box 1) in mesenchymal progenitors (Kobayashi et al., 2004; Schnabel et al., 2003; Guioli et al., 2007; Orvis and Behringer, 2007; Ayers et al., 2015; Jacob et al., 1999; Atsuta and Takahashi, 2016; Prunskaite-Hyyryläinen et al., 2016; Vainio et al., 1999; Coffinier et al., 1999; Lokmane et al., 2010). A subset of epithelial progenitors also expresses the receptor for the anti-Müllerian hormone, Misr2 (also known as Amhr2; Box 1) (Ghosh et al., 2020). These Pax and homeobox members constitute an expression signature conserved among the epithelial lineages of the urogenital system, suggesting a role as master regulators controlling urogenital developmental programmes with potential co-regulation and cooperation (Pedersen et al., 2005; Bouchard et al., 2002, 2004; Torres et al., 1995; Guioli et al., 2007; Vainio et al., 1999; Carroll et al., 2005; Barnes et al., 1994; Kobayashi et al., 2004; Davis et al., 2008; Miyamoto et al., 1997; Pellegrini et al., 1997; Grote et al., 2008). Furthermore, Müllerian progenitors also upregulate secreted proteins from the Wnt family (Box 1), such as Wnt7a in the epithelial precursors and Wnt4 in the mesenchymal ones (Vainio et al., 1999; Miller et al., 1998; Kobayashi et al., 2004). Expression of the Dach1/2 (Box 1) transcriptional cofactors has also been reported in epithelial progenitors during this initial specification stage (Davis et al., 2008).

Several observations, predominantly in chicken and mouse models, have identified the GRNs that orchestrate the Müllerian progenitors' specification and their interaction with conserved embryonic signalling pathways. Atsuta and Takahashi (2016) reported Pax2 as the first transcriptional regulator expressed in the Müllerian ridge in chicken embryos and showed that its expression is regulated by the BMP pathway. Pax2, along with the FGF/ERK/MAPK pathway, regulate Lim1 expression in the Müllerian ducts. Downregulation of any of these factors and the intracellular modulator of the fibroblast growth factor (FGF) pathway (Box 1), Ras, results in Lim1 downregulation. Interestingly, the same study also reported crosstalk between the BMP and FGF/RAS/MAPK pathways (Atsuta and Takahashi, 2016). Moreover, Lim1 and Wnt7a expression is severely affected in the Müllerian ducts of *Dach2^−/−^; Dach1^+/−^* and *Dach2^+/−^; Dach1^−/−^* murine females, although the underlying mechanisms remain undetermined (Davis et al., 2008).

An important feature of early Müllerian development is the mesoepithelial nature of the epithelial lineage progenitors. This persists in the Müllerian ducts until differentiation into a committed epithelial phenotype at post-Müllerian developmental stages. These cellular dynamics suggest that coelomic epithelial progenitors give rise to the mesoepithelial lineage through a partial epithelial-mesenchymal transition (EMT; Box 1) and to the mesenchymal lineage through full EMT (Atsuta and Takahashi, 2016; Jacob et al., 1999; Dohr and Tarmann, 1984). Later, the mesoepithelial lineage differentiates into epithelium through the opposite mechanism, mesoepithelial-epithelial transition (MOET) (Jacob et al., 1999; Dohr and Tarmann, 1984; Orvis and Behringer, 2007). EMT is observed during Müllerian progenitors' specification when the coelomic epithelial basal membrane ruptures (Atsuta and Takahashi, 2016), whereas MOET is detected by the gradual increase in E-cadherin expression after Müllerian development and the acquired apicobasal polarity and epithelial morphology before birth (Orvis and Behringer, 2007; Stewart et al., 2013; Ghosh et al., 2017; Prunskaite-Hyyryläinen et al., 2016).

Following this initial stage, the Müllerian-committed coelomic progenitors invaginate into the underlying mesonephric stroma. The Müllerian ridge is a contractile structure that bends by transmitting tension across the underlying epithelial sheets. The FGF/RAS/MAPK pathway seems to play a role during apical constriction, as downregulation of *Fgfr2* and *Rac1* impairs Müllerian invagination (Atsuta and Takahashi, 2016). Furthermore, the invagination of Müllerian mesoepithelial progenitors is controlled by the endogenous expression of Lim1 (also known as Lhx1) and the mesenchymal progenitors' expression of Wnt4 (Kobayashi et al., 2004; Atsuta and Takahashi, 2016; Orvis and Behringer, 2007; Vainio et al., 1999). This evidence is supported by Lim1 and Wnt4 loss-of-function mouse and chicken models (Kobayashi et al., 2004; Kobayashi and Behringer, 2003; Prunskaite-Hyyryläinen et al., 2016; Guioli et al., 2007; Vainio et al., 1999). Moreover, the phosphoinositide 3-kinase (PI3K) pathway (Box 1) is activated during this phase (Fujino et al., 2009). The end of this second stage is marked by the invaginating Müllerian tip physically contacting the adjoining Wolffian duct (Gruenwald, 1941; Guioli et al., 2007; Orvis and Behringer, 2007; Hashimoto, 2003; Kobayashi et al., 2004; Jacob et al., 1999; Dohr and Tarmann, 1984).

The growing Müllerian duct finalises its formation by elongating along the mesonephros in intimate contact with the Wolffian duct. A high cell proliferation rate is the predominant mechanism in this final stage and occurs along both the Müllerian anteroposterior and dorsoventral axes, elongating and thickening the forming tubes (Kobayashi et al., 2004; Orvis and Behringer, 2007; Guioli et al., 2007; Atsuta and Takahashi, 2016; Gruenwald, 1941; Jacob et al., 1999; Fujino et al., 2009). The physical contact between the Müllerian tip and the Wolffian epithelial duct establishes a new dependency. The Wolffian duct controls Müllerian elongation by expressing Wnt9b and the master regulator Gata3 (Box 1), although the physical interaction has been suggested to play a role too (Carroll et al., 2005; Grote et al., 2008; Hashimoto, 2003; Dohr and Tarmann, 1984; Gruenwald, 1941). Wnt9b controls Müllerian elongation by activating the canonical Wnt pathway (Carroll et al., 2005), whereas reduced Gata3 expression arrests elongation at later embryonic stages (Grote et al., 2008). Wnt4 and Dmrt1, expressed in mesenchymal cells and the coelomic epithelium, also play a role in the elongation (Kobayashi et al., 2005; Prunskaite-Hyyryläinen et al., 2016; Ayers et al., 2015). If either are downregulated, the mesenchymal cells do not migrate or attach to the elongating tube, and development is truncated (Prunskaite-Hyyryläinen et al., 2016; Ayers et al., 2015). Despite the important role of the Wolffian epithelium in this developmental stage, the contribution of cells from the Wolffian duct to the growing Müllerian tube has been widely refuted (Guioli et al., 2007; Orvis and Behringer, 2007; Gruenwald, 1941; Kobayashi et al., 2005; Carroll et al., 2005). Moreover, the adhesion G protein-coupled receptor Gpr56 (also known as Adgrg1; Box 1) expressed in mesoepithelial progenitors from early developmental stages and the PI3K/Akt pathway play a role as well. Decreased Gpr56 leads to a reduction in the number of proliferating cells *in vivo* and *in vitro* and impairs elongation (Roly et al., 2020), and disruption of PI3K/Akt signalling arrests elongation, as the PI3K/Akt pathway seems to display an antiapoptotic role (Fujino et al., 2009). Finally, after the elongation stage, human Müllerian ducts and the urogenital sinus create the female reproductive tract primordium. The Müllerian segments corresponding to the uterus, cervix and the upper two-thirds of the vagina fuse, and, subsequently, the midline fused sections resorb to shape a unique uterine cavity and a canalised cervix and vagina. Postnatally, transcriptional programmes prompting the differentiation of Müllerian sections into the mature female reproductive organs are activated (Ghosh et al., 2017; Yamanouchi et al., 2010; Komatsu and Fujita, 1978; Kurita et al., 2001).

## Disruption of Müllerian developmental and differentiation processes

### Classification of MDAs

**Fig. 2.**
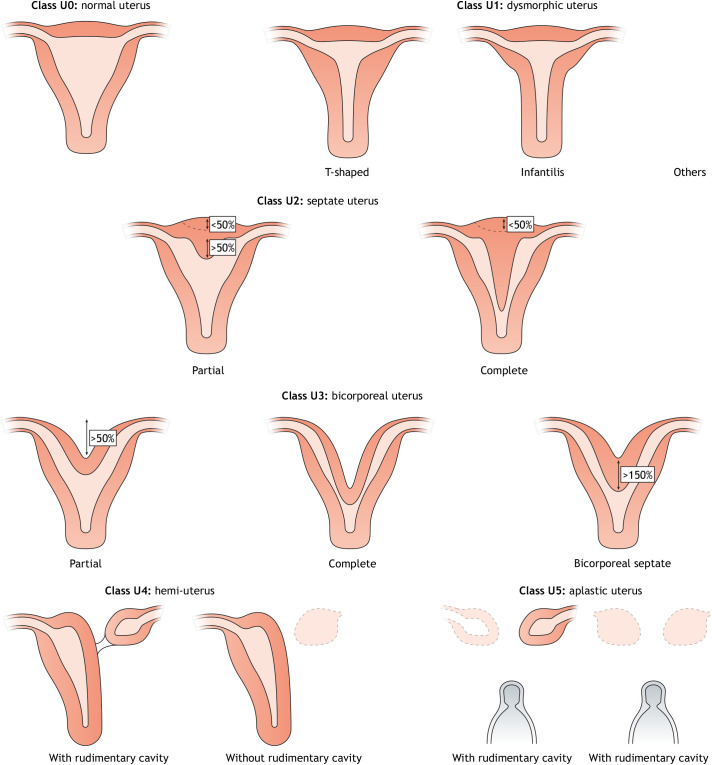
**The ESHRE/ESGE consensus classification system of female reproductive anomalies organises uterine malformations based on severity.** Adapted from Grimbizis et al. (2013), under the terms of the CC-BY 3.0 license.

Disturbances during Müllerian duct development and differentiation result in a wide spectrum of MDAs. Although several classifications of MDAs exist (Buttram and Gibbons, 1979; AFS, 1988; Acién and Acién, 2011; Oppelt et al., 2005), we will follow the European Society of Human Reproduction and Embryology (ESHRE) and European Society for Gynaecological Endoscopy (ESGE) classifications in this Review. In 2013, ESHRE/ESGE created a joint working group, Congenital Uterine Anomalies (CONUTA), which introduced a consensus classification system of female reproductive anomalies (Grimbizis et al., 2013). The ESHRE/ESGE method systematically organises Müllerian malformations into five main U classes and a-c subclasses determined by uterine abnormalities and the stage of embryonic development at which they occurred Fig. 2; Box 2). This classification naturally follows an inverse chronological developmental order with higher-class disorders prompted by changes at earlier developmental stages. Müllerian organogenesis, fusion and septal resorption aberrancies correlate with Class 4 and 5, Class 3 and Class 2 MDAs, respectively, whereas Class 1 is likely to derive from Müllerian differentiation anomalies. To complement the uterine categorisation (Box 2), the classification also includes co-existent classes based on additional cervical and vaginal anomalies (C and V classes), thus favouring an exhaustive definition of the usually multiorgan heterogeneous defects.

**Box 2. The ESHRE/ESGE system for MDA classification**

This system comprises the main classes that align with the inverse chronological developmental order of the female reproductive tract.

• Class 0, normal uterus: defines a normal uterus and includes disorders in which only the cervix or vagina are affected.

• Class 1, dysmorphic uterus: describes congenital malformations of the uterine cavity, excluding septa.

• Class 2, septate uterus: incorporates anomalies affecting a normal septal resorption in disorders of incomplete midline fusion of the Müllerian ducts.

• Class 3, bicorporeal uterus: includes disorders arising from abnormal fusion of the convergent Müllerian ducts. Subclass c of this category includes cases with additional septal resorption defects. Furthermore, this class includes complete bicorporeal uterus, a Class C2 disorder exhibiting a double cervix, which was traditionally termed didelphys uterus. The use of this term is currently discouraged.

• Class 4, hemi-uterus: this class describes malformations arising from a partial Müllerian developmental impairment in which only one tube fully develops whereas the other one is partially or completely absent.

• Class 5, aplastic uterus: this category describes a more severe version of Class 4 disorders, featuring a complete absence or severe underdevelopment of the Müllerian tubes. In some cases, it can present a bi/unilateral rudimentary horn with or without a uterine cavity.

• Class 6, unclassified cases: every malformation and combined pathologies or disorders that do not fit within the Classes 0-5.

Within the main U classes, subclasses and co-existent C and V classes, anomalies are organised following increasing severity, apart from main U class 6 (Box 2), which is reserved to malformations that do not fit into the other classes. However, this classification system could have benefitted from an extra co-existent class describing fallopian tube deformities to cover the whole female reproductive tract.

### Müllerian duct developmental syndromes

#### Mayer–Rokitansky–Küster–Hauser syndrome

**Fig. 3.**
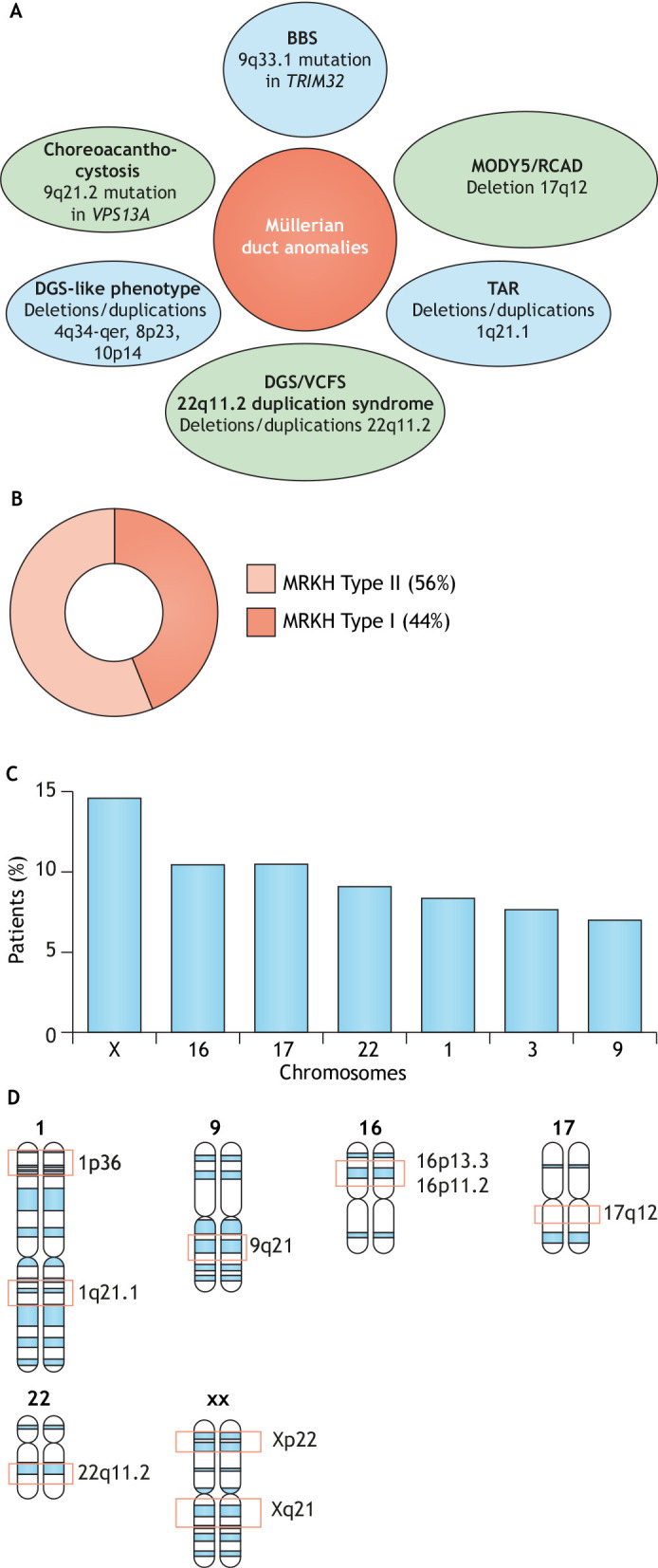
**Shared and unique MDA genomic lesions and incidence.** (A) Summary of genetic lesions that MDAs share with other ‘unrelated’ syndromes. BBS, Bardet–Biedl syndrome; DGS, DiGeorge syndrome; MODY5, maturity-onset diabetes of the young, type 5; RCAD, renal cyst and diabetes syndrome; TAR, thrombocytopenia-absent radius syndrome; VCFS, velocardiofacial syndrome. (B) Incidence of Mayer–Rokiransky–Küster–Hauser (MRKH) types. (C) Chromosomes that are recurrently affected by rearrangements and CNVs in MRKH and percentage of patients exhibiting lesions in each chromosome. Computed from a dataset of 144 patients compiled from the published studies summarised in Table S1. (D) Chromosomal regions in which recurrent lesions occur.

MDAs genetically overlap with several seemingly unrelated syndromes (Fig. 3A). Mayer–Rokitansky–Küster–Hauser (MRKH) syndrome (OMIM 277000) is a congenital MDA characterised by aplasia of some Müllerian-derived reproductive structures, including the uterus, cervix and upper vagina, in women with a normal secondary sexual development and a 46, XX karyotype (Morcel et al., 2007; Fontana et al., 2017; Guerrier et al., 2006). It falls into Class 5 of the ESHRE/ESGE classification (Box 2), accounting for a high degree of severity (Grimbizis et al., 2013). Even though Müllerian aplasia manifests in many MDAs, 90% of the clinical cases showing this feature are diagnosed as MRKH, and the incidence of this condition is ∼1 in 4500-5000 female newborns. MRKH typically presents with cervicovaginal absence and either an absent, bicornuate or rudimentary uterus (Morcel et al., 2007; Fontana et al., 2017; Guerrier et al., 2006; Herlin et al., 2020). The fallopian tubes and ovaries generally appear normal, with no endocrine abnormalities, although abnormal tubes and ovaries or tubal agenesis (Box 1) have occasionally been reported (Cheroki et al., 2007; Devriendt et al., 1997; Bernardini et al., 2009; Nik-Zainal et al., 2011; Oppelt et al., 2012; Rall et al., 2015).

The MRKH syndrome is classified into Type I if the reproductive system is exclusively disturbed or Type II if additional systems are affected. The Type I disorders, also known as typical, isolated or Rokitansky sequence, account for 44% of MRKH cases (Strübbe et al., 1994, 1992). Patients with Type I MRKH are usually diagnosed during late adolescence due to primary amenorrhoea, although they are usually asymptomatic and their secondary sexual development is normal. The Type II or associated disorders account for 56% of MRKH cases (Fig. 3B) (Strübbe et al., 1992), although in patients of Chinese ethnicity only 7.2% of MRKH cases were Type II (Pan and Luo, 2016). In addition to the general Müllerian-associated features, MRKH Type II can feature further aberrations typically related to the renal and spinal systems. The most severe manifestation of MRKH type II is the syndrome Müllerian duct aplasia, unilateral renal agenesis and cervicothoracic somite anomalies (MURSC; OMIM 601076) (Strübbe et al., 1992; Duncan et al., 2020). It is detected in ∼19% of MRKH type II patients (Strübbe et al., 1992). Additionally, heart malformations and hearing impairment can rarely be observed in MRKH Type II (Morcel et al., 2007; Fontana et al., 2017). The concomitant aberrations of Müllerian ducts and different organs in MRKH Type II suggest a perturbance of conserved master molecular pathways orchestrating organogenesis or chromosomal rearrangements. A form of MRKH exhibiting the general utero-cervix-vaginal atresia (Box 1) presentation has been linked to hyperandrogenism (OMIM 158330) (Biason-Lauber et al., 2004, 2007; Philibert et al., 2008, 2011; Chang et al., 2012).

#### Other MDA syndromes

Herlyn–Werner–Wunderlich syndrome (HWWS; OMIM 192050) is a rare disorder presenting a variable clinical picture with diverse triad combinations of urogenital aberrations, such as uterine malformations, unilateral cervicovaginal obstruction and ipsilateral renal anomalies. Among the uterine malformations, bicorporeal uterus and double cervix occur in 62.5% of patients, septate uterus in 22.5% and bicornuate uterus in 15%. Ipsilateral vaginal septum was found in 75% of patients and ipsilateral cervical obstruction in 25% (Zhang et al., 2020). Ipsilateral renal agenesis, as well as ipsilateral paravaginal cysts and ureter remnants invading the obstructed vagina/cervix have also been reported (Zhang et al., 2020; Aveiro et al., 2011). The incidence of HWWS in the population is unknown, although a study reported 0.1-3.8% (Aveiro et al., 2011).

Hand-foot-genital syndrome (HFGS; OMIM 140000) is an uncommon syndrome with an autosomal-dominant inheritance pattern (Box 1). Clinical presentations are dominated by urogenital and limb malformations. Defects in the female reproductive system typically encompass aberrant Müllerian fusion, giving rise to different versions of bicorporeal uterus abnormalities (Class U3) (Mortlock and Innis, 1997; Goodman et al., 2000).

Regardless of which form they take, MDAs are congenital disorders of genetic/genomic origin. Owing to the complex inheritance and phenotypic variability within the MDA clinical spectrum, establishing an inheritance pattern and a phenotype-genotype correlation has been challenging even for the same genomic/genetic lesion. The recent progress in several technological fields, model systems and patient-based research could help to improve this situation.

## Technologies and strategies to investigate MDA genetics and genomics

### Knowledge from conventional approaches

Screening for the genomic and genetic aberrations underpinning MDA aetiology and pathogenesis have helped in shaping their syndromic landscape. However, discordant phenotypes in monozygotic twins casts doubt on the purely genetic aetiology of MDAs (Regenstein and Berkeley, 1991; Steinkampf et al., 2003; Duru and Laufer, 2009; Milsom et al., 2015). Both *de novo* and familial MDA cases have been documented, with familial ones following an autosomal-dominant inheritance pattern with reduced penetrance and variable expressivity (Box 1) (Cheroki et al., 2007; Devriendt et al., 1997; Jacquinet et al., 2020; Bendavid et al., 2007; Williams et al., 2016; Bernardini et al., 2009; Hinkes et al., 2012; Morcel et al., 2011; McGowan et al., 2015; Uliana et al., 2008; Cheroki et al., 2006; Rall et al., 2015; Ledig et al., 2018; Pan et al., 2019). These findings highlight the complex nature of MDAs and suggest a multifactorial causation and phenotypic expression.

Despite the challenges that these developmental disorders pose, researchers have identified pathological factors such as *de novo* and inherited genomic structural variations, mutations, mosaicisms and epigenetic aberrations as underlying MDAs. The majority of MDA investigations have traditionally benefitted from three strategies. First, genomic studies looking for chromosomal aberrations in MDA patients followed by identification of candidate genes mapping within the lesions (Table S1). Second, mutational screening in the normal and MDA-affected population. Third, causation testing in animal models. Molecular techniques such as array comparative genomic hybridisation (aCGH) and single-nucleotide polymorphism (SNP) microarrays, multiplex ligation-dependent probe amplification (MPLA; Box 1), FISH, polymerase chain reaction (PCR) and quantitative PCR (qPCR), Sanger sequencing and duplex PCR/liquid chromatography (DP/LC; Box 1) have enabled this progress. They are the most frequently used for exploratory studies as well as for validation, individually and in combination.

Numerous human genetic anomalies are linked to rearrangements at the genomic level, which prompt an overall gain, loss or translocation of genetic material, resulting in CNVs. Around 10-58% of patients with MDAs bear such aberrations (Table 1). These genomic imbalances have been detected in all chromosomes; however, recurrent ones are observed on chromosomes X, 16, 17, 22, 1, 3, 9 in decreasing order of frequency (Fig. 3C,D). Most lesions in MDAs account for deletions and duplications, yet translocations have also been described (Kucheria et al., 1988; Williams et al., 2016). Many of these lesions are shared with other syndromes; hence, MDAs are commonly part of a broader clinical spectrum that superficially unrelated diseases present (Fig. 3A).

**Table 1.**
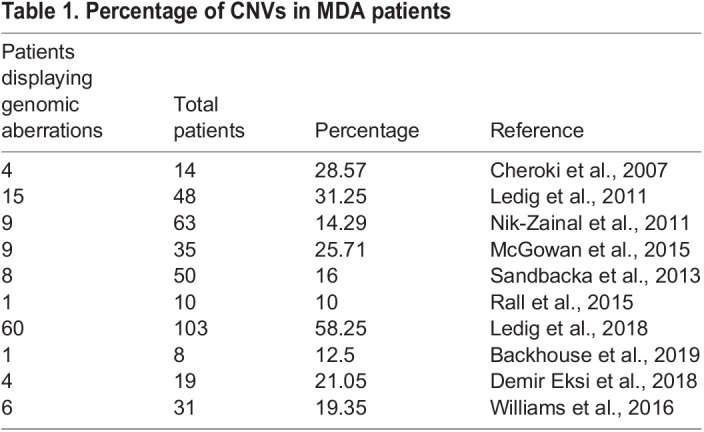
Percentage of CNVs in MDA patients

Aberrations in chromosome 17 are homogeneous among clinical MDA cases and span a 1.2-1.9 Mb deletion in 17q12 (Table S1 and references therein). This genomic region harbours the *LHX1* (Bozzi et al., 1996) and *HNF1B* genes that encode the transcription factors LIM1 and HNF1β, respectively. Mutational screening for these genes in MDA patients with no 17q12 deletion identified a heterozygous frameshift mutation in *LHX1* resulting in a premature stop codon, as well as missense mutations (Bernardini et al., 2009; Ledig et al., 2011, 2012; Sandbacka et al., 2013). As previously discussed, LIM1 is part of the early Müllerian duct development GRN and is essential for Müllerian invagination. Müllerian progenitors in female mice harbouring a null mutation in *Lim1* do not invaginate (Kobayashi et al., 2004), which is the same phenotype observed in chicken embryos following *Lim1* downregulation (Atsuta and Takahashi, 2016). Mouse models with homozygous mutations in *Hnf1b* lack Müllerian ducts (Lokmane et al., 2010). *HNF1B* mutations were also found in patients with bicorporeal uterus and bicorporeal uterus with double cervix (Bingham et al., 2002). Loss-of-function deletions in *HNF1B* triggering maturity-onset diabetes of the young, type 5 [MODY5; also known as renal cyst and diabetes syndrome (RCAD); OMIM 137920; Box 1] have also been reported to cause Müllerian phenotypes in two patients (Lindner et al., 1999). The recurrent 17q12 deletion is present in MODY5 as well. Only *HNF1B* was investigated in Bingham et al. (2002) and Lindner et al. (1999), and whether a 17q12 deletion spanning *LXH1* or whether a mutation in this gene contributed to the reported phenotypes is an open question. Furthermore, patients with RCAD (Box 1) also occasionally display MDAs related to 17q12 deletions (Cheroki et al., 2007).

Aberrations in chromosome 22 have also been reported in MDA patients and include deletions or duplications, mainly at 22q11.2 (Sundaram et al., 2007; Devriendt et al., 1997; Ledig et al., 2011, 2018; Morcel et al., 2011; Uliana et al., 2008; Cheroki et al., 2006). Some patients suffering from DiGeorge syndrome (22q11.2 deletion syndrome; Box 1) additionally display congenital MDA/MRKH phenotypes, although the causative genes related to the urogenital anomalies within this chromosome are currently unknown (Cheroki et al., 2007; Sundaram et al., 2007; Devriendt et al., 1997; Ledig et al., 2011; Morcel et al., 2011; Nik-Zainal et al., 2011). Aberrations in chromosome 1 in patients with MDAs include deletions/duplications of variable size, mainly at 1q21.1 (Ledig et al., 2011; McGowan et al., 2015; Demir Eksi et al., 2018; Chen et al., 2015). This region contains the candidate MDA gene *RBM8A* (Box 1), as four variants were found to be associated with MDAs (Tewes et al., 2015). Some patients diagnosed with thrombocytopenia-absent radius syndrome (TAR; OMIM 27400; Box 1) also present Müllerian abnormalities and 1q21.1 CNVs (Ledig et al., 2011; Behera et al., 2005; Griesinger et al., 2005). MDA-related lesions in the X chromosome are heterogeneous (Cheroki et al., 2007; Ledig et al., 2011, 2018; McGowan et al., 2015; Demir Eksi et al., 2018). *KLHL4* (Box 1) resides in this region and has also been proposed as a causative gene in Müllerian abnormalities, given that a deletion at Xq21.32 uniquely spanning *KLHL4* was the only aberration observed in an MDA case report (Cheroki et al., 2007). Lesions on chromosome 16p11.2 are homogeneous, ranging from 0.55 Mb to 0.6 Mb (Bendavid et al., 2007; Ledig et al., 2011; Morcel et al., 2011; McGowan et al., 2015; Ledig et al., 2018), and include *TBX6* (Box 1), which has been proposed as causative of the Müllerian phenotype. Splicing and missense mutations (Waschk et al., 2016; Sandbacka et al., 2013), as well as two missense SNPs in exons 4 and 6 of *TBX6*, were more frequently observed in MDA patients compared with healthy controls (Sandbacka et al., 2013). Furthermore, the *TBX6* variants c.1015C>A and c.484G>A were found in six MDA patients (Tewes et al., 2015). Animal models with mutations in this gene display skeletal malformations that are reminiscent of the MRKH type II phenotype, although urogenital malformations have not been investigated in these models (White et al., 2003).

Additional genes involved in embryonic development of the genital system have been investigated in patients with MDAs. For instance, some patients with MRKH associated with hyperandrogenism bear mutations in *WNT4* (Biason-Lauber et al., 2004, 2007; Philibert et al., 2008, 2011), although mutational analysis of MRKH women without hyperandrogenism did not identify disruptions in this gene (Ravel et al., 2009; Chang et al., 2012). The *WNT4* case reports did not comprehensively study other genomic aberrations these patients might carry, so it is unknown whether mutant *WNT4* is the only contributor to the MRKH/hyperandrogenism phenotype. Moreover, some patients with MRKH and hyperandrogenism lacked mutations in *WNT4*, suggesting that other components of the WNT pathway might be altered or that other pathways might be responsible (Philibert et al., 2011; Chang et al., 2012). Animal models show that Wnt4 is essential for female development, as it is required for Müllerian invagination and because it suppresses Leydig cell (Box 1) development in the gonad (Vainio et al., 1999). In a mouse model bearing a mutated *Wnt4*, Müllerian invagination does not occur and testosterone is synthesised (Vainio et al., 1999). Nevertheless, *Lim1* and *Wnt7a* expression in mesenchymal progenitors is conserved, indicating that Müllerian progenitor specification still occurs in this model (Orvis and Behringer, 2007; Kobayashi et al., 2004; Vainio et al., 1999; Prunskaite-Hyyryläinen et al., 2016). Moreover, downregulation of *Wnt4* in chicken embryos impaired Müllerian elongation due to lack of mesenchymal cell traffic to generate the Müllerian ducts, which is the same phenotype presented when *Dmrt1* is downregulated (Prunskaite-Hyyryläinen et al., 2016; Ayers et al., 2015).

Pax2 and Pax8 belong to the group of core transcriptional factors specific to the urogenital epithelial lineages (Pax2, Pax8, Emx2, Lim1, Pbx1 and Hnf1b). Most animal models harbouring mutations in these factors typically show aberrations at early stages of Müllerian development. A human *PAX2* polymorphism (SNP rs12266644, G>T) has been significantly linked to MDAs in one study (Xu et al., 2017), although a previous mutational analysis in 192 patients with Müllerian abnormalities only identified a synonymous *PAX2* mutation (c.320G>A) in one patient with complete bicorporeal uterus and a double cervix (U3bC2) (Wang et al., 2012). Moreover, individuals with septate uterus had *PAX2* hypomethylation and increased expression levels (Wang et al., 2020). In Pax2-null mouse models, Müllerian development is blocked soon after invagination due to the lack of Wolffian ducts (Kobayashi et al., 2004; Torres et al., 1995). This contrasts with the phenotype in chickens, in which *Pax2* downregulation fully impairs invagination (Atsuta and Takahashi, 2016). In chicken, abrogating *Pax2* expression almost completely depletes *Lim1* expression; however, in the Pax2-deficient murine model, *Lim1* is still upregulated in Müllerian mesoepithelial progenitors (Kobayashi et al., 2004; Torres et al., 1995; Atsuta and Takahashi, 2016). This explains the phenotypical dichotomy in *Pax2* model systems, although additional findings can explain this phenomenon further. The *Pax2^−/−^*/*Pax8^+/−^* mouse line has substantially decreased levels of *Lim1* mRNA in the mesonephros, which suggests that Pax2/8 exert functional redundancy (Boualia et al., 2013). This redundancy has been previously shown in renal system development (Bouchard et al., 2002). Furthermore, the chicken phenotype can be explained by the absence of *Pax8* in the genome. Interestingly, *Pax8-*null mouse lines do not display Müllerian aberrations (Vainio et al., 1999; Carroll et al., 2005; Bouchard et al., 2004).

Additionally, no Müllerian structure forms in mice lacking *Emx2* and *Pbx1* (Miyamoto et al., 1997; Schnabel et al., 2003). A study of four MRKH patients did not find any lesions in *PBX1* (Burel et al., 2006). However, a subsequent study found a susceptibility SNP in *PBX1* (rs2275558) associated with the MRKH syndrome (Ma et al., 2015). A nonsense mutation in *EMX2* has been reported in a patient with complete bicorporeal uterus exhibiting a double cervix (Liu et al., 2015). Uterine expression of *EMX2* was also increased in patients displaying a partially septate uterus (Zhu et al., 2016). Furthermore, the Hox A homeobox genes (Box 1) have been investigated due to their roles in Müllerian duct differentiation. The 5′-end genes of the Hox A cluster are important for female reproductive function and are expressed in the female reproductive tract following this spatial sequence: *Hoxa9* in the fallopian tubes, *Hoxa10* in the uterus, *Hoxa11* in the lower uterus and endocervix, and *Hoxa13* in the ectocervix and upper vagina (Taylor et al., 1997; Ma et al., 1998). The search of mutations in *HOXA7* and *HOXA9-HOXA13* in MDA patients was unsuccessful in some studies (Burel et al., 2006; Liatsikos et al., 2010; Chen et al., 2014), although others have reported interesting causative findings. *HOXA5* and *HOXA9* were overexpressed and hypomethylated in MRKH patients (Rall et al., 2011), and nonsense and missense *HOXA13* mutations, as well as an expansion of the HOXA13 N-terminal polyalanine tract, have been reported as causative of HFGS (Mortlock and Innis, 1997; Goodman et al., 2000). Furthermore, the genomic localisation of *HOXA13* 7p15.2 is very interesting due to the MRKH cases that report aberrations in 7p14.3 and 7p14.1 (Ledig et al., 2011; Rall et al., 2015). The expression of *HOXA10* was downregulated in the endometrium of MDA patients (Zhu et al., 2016), and pathogenic mutations in *HOXA10* have been linked to women with MDAs (Ekici et al., 2013). Also, a causative mutation in *HOXA11* was found in a patient with septate uterus (Zhu et al., 2017). The functions of Hox genes have also been extensively investigated using mouse models. Although no Müllerian developmental defect has been reported in *Hoxa9*-null mice (Raines et al., 2013; Fromental-Ramain et al., 1996), *Hoxa10*- and *Hoxa11-*knockout mice display anterior homeotic transformations in the reproductive tracts and *Hoxa13*-mutant mice do not develop the caudal portion of the Müllerian ducts (Satokata et al., 1995; Benson et al., 1996; Gendron et al., 1997; Warot et al., 1997).

Wnt7a, Wnt9b and Wnt5a are Wnt family members essential for Müllerian development. Although no *WNT7A* mutations were found in women bearing MDAs (Ravel et al., 2009; Timmreck et al., 2003; Dang et al., 2012), homozygous *WNT7A* mutations are the known underlying cause of Al-Awadi/Raas-Rothschild/Schinzel phocomelia syndrome (OMIM 276820; Box 1), which produces skeletal and Müllerian defects as well as occipital meningocele (Al-Qattan, 2013). Mouse lines lacking Wnt7a do not display disorders during Müllerian development but at later differentiation stages (Parr and Mcmahon, 1998; Miller et al., 1998). A 3′ untranslated region variant, a nonsense, a missense and five likely pathogenic mutations in *WNT9B* have been reported to date in women bearing MDAs (Waschk et al., 2016; Wang et al., 2014). However, a study of 542 Chinese patients excluded *WNT9B* as a causative gene for MDAs (Tang et al., 2014), although one study found a WNT9B polymorphism (rs34072914) to be highly associated with MRKH syndrome risk (Ma et al., 2015). Knocking out *Wnt9b* in mice blocks Müllerian elongation (Carroll et al., 2005). This phenotype is shared with embryos with conditional *Lim1* knockout specific to the Wolffian ducts. In this mouse line, Wnt9b expression is significantly reduced, suggesting that Wnt9b is a downstream target of Lim1 (Pedersen et al., 2005)*.* Although Wnt5a plays a central role in Müllerian duct development (Mericskay et al., 2004), a survey of WNT genes in 11 MRKH patients did not find any mutations in *WNT5A* (Ravel et al., 2009).

Besides those that directly influence Müllerian development, additional genes have been linked to MDAs. Partial duplication of the *SHOX* gene, which encodes a transcription factor linked to bone and cartilage development, has been found in some MRKH Type I patients (Gervasini et al., 2010). *SHOX* is a homeobox gene that is highly conserved among different species but absent from the mouse genome (Marchini et al., 2016). *SHOX* mutations cause skeletal abnormalities in chicken embryos resembling MRKH Type II-associated aberrations, although MDAs have not been reported in this model (Tiecke et al., 2006). Missense mutations in the sexual hormone receptors OXTR and ESR1 have also been found in MRKH patients (Brucker et al., 2017). Furthermore, cystic fibrosis transmembrane conductance regulator gene (*CFTR*) mutations were found in patients affected by Müllerian agenesis (Timmreck et al., 2003).

A combined SNP microarray and aCGH study of MRKH patients identified 42 homozygously altered genomic regions, two of them spanning the 22q11.21 locus known for recurrent CNVs in MDAs. Other regions also spanned putative MDA-related genes such as *RBM8A*, *CMTM7*, *CCR4*, *TRIM71*, *CNOT10*, *TP63*, *EMX2* and *CFTR* (Demir Eksi et al., 2018).

Importantly, several animal models identified loss-of-function mutations that induce Müllerian anomalies but have not been investigated or reported to be associated with human MDAs yet. Among these are genes involved in retinoic acid (RA) signalling. RA signalling is transduced by RA receptors (RARs) and RXRs (retinoid X receptors). Single RAR mutations in mouse models such as those in *Rara1*, *Rara2*, *Rarb2* or *Rarg*, have not been found to be causative of developmental disorders. However, compound mutations in these genes display a mutant phenotype. *Rara^−/−^/Rarb2^−/−^* knockouts arrest Müllerian elongation in a Wolffian-independent manner, whereas *Rara1^−/−^/Rarb2^−/−^* and *Rara^−/−^/Rarg^−/−^* mice present with late arrest of Müllerian elongation owing to the loss of the Wolffian caudal end (Mendelsohn et al., 1994). Also, an *Rxra^−/−^/Rara^−/−^* genotype leads to a loss of Müllerian ducts (Kastner et al., 1997). However, a study of 25 women with MRKH did not find any mutations in *RARG* and *RXRA* (Cheroki et al., 2006). In another example, mice with co-occurring homozygous mutations in *Dach1*/*2*, which encode chromatin-associated transcription regulators, result in MDAs that are independent of Wolffian development, as the male tubes develop and differentiate normally. Dach1-null mice, Dach2-null mice and double-heterozygous mutants display a normal phenotype (Davis et al., 2008), which suggests functional redundancy and compensation among Dach1/2. Lastly, large tumour suppressor kinase 1/2 (Lats1/2) are serine/threonine-specific protein kinases that belong to the Hippo pathway. They inactivate the Yap and Taz cofactors, and their conditional deletion in mouse models increases the expression of Yap, Taz and Ctgf in the Müllerian mesenchyme. This results in a myofibroblast fate specification of mesenchymal pluripotent stem cells and malformations in the Müllerian duct (St-Jean et al., 2019). To the best of our knowledge, no studies in human MDA patients identified alterations in *DACH1/2* and *LATS1/2*.

### The omics era, the organoids revolution and genome-wide databases

The thriving development of novel, high-throughput and unbiased genome-wide technologies in recent years has broadened our knowledge and allowed for substantial progress in the field of rare congenital diseases. These scientific breakthroughs have prompted the adaptations of new approaches to the unbiased study of Müllerian duct diseases. This section reviews the new insights into MDAs they have provided so far.

In the current zenith of NGS tools, whole-genome sequencing (WGS; Box 1), whole-exome sequencing (WES), RNA sequencing (RNA-seq) and their respective single-cell versions hold the promise of boosting the discoveries and associations in the field of MDA aetiology. WGS achieves unbiased high-resolution analysis of the coding and non-coding genome. A single WGS experiment can simultaneously detect distinct structural variations in the coding and non-coding sections of the genome as well as in mitochondrial DNA, whereas WES accounts for an unbiased comprehensive interrogation of the coding genome for identifying candidate genes that contribute to MDA pathogenesis. These techniques may suitably complement and be validated by the traditional approaches summarised in the previous sections. Additionally, these bulk genomic assays can be used in combination with complementary single-cell transcriptomics and epigenomics (Clark et al., 2016), as well as with model organisms and 3D organoid models (Box 1) mimicking the healthy and pathological organogenesis of the Müllerian ducts (Alzamil et al., 2020). Single-cell transcriptomics and epigenomics could potentially identify the cell subtypes and populations accountable for the embryonic wild-type and diseased Müllerian ducts and derived adult organs. Furthermore, they could accelerate the discovery of new gene regulatory networks in these organs at unprecedented resolution, which could expand the currently known repertoire of genetic regulators and increase the discovery of new genetic alterations that can cause MDAs. Two recent examples identified the single-cell transcriptional landscape of the MRKH uterus (Hentrich et al., 2020) and of the embryonic Müllerian duct of the chicken (Roly et al., 2020). Mucenski et al. (2019) adopted a similar approach, using single-cell RNA-seq of Hox wild-type and mutant mouse uteri to define the populations of both states, and identified the Wnt and the Cxcl12/Cxcr4 pathways as significantly disrupted during Hox absence, confirming that genetic aberrations can have complex cellular consequences. Integrating novel and conventional strategies and models holds the promise to deciphering the multifactorial basis underlying heterogeneous MDAs (Posey, 2019).

To date, a single WGS study of nine patients with MRKH type I and their respective parents has been conducted (Pan et al., 2019). It identified *de novo* missense mutations in *TNK2*, *PIK3CD* and *SLC4A10* predicted to be deleterious and 623 *de novo* SNVs in non-coding regions, of which some mapped to DNase1-hypersensitive sites (Box 1), transcription factor binding sites, enhancer elements and pseudogenes. Twenty-nine variants were recurrent. Two of them (chr1: 145291364 A>T and chr1: 145291369 G>A) map to the 3′ untranslated region of *NOTCH2NL* (also known as *NOTCH2NLA*), which also functions as the promoter of *NBPF10*. Furthermore, this study detected 39 *de novo* structural variants such as deletions, tandem duplications, inter-chromosomal translocations and inversions. CNVs were found in chromosomes 1, 2, 3, 7, 8, 10, 11, 13, 14, 15, 16, 17, 20 and 22, and a deletion at 15q11.2 spanning *LOC727924*, *OR4N4* and *OR4M2* genes was identified in all nine families. This deletion has previously been documented in three patients with MRKH Type I (Chen et al., 2015). Furthermore, *KRTAP5-10* and three enhancer regions for *DUOX1*, *MLLT4* (also known as *AFDN*) and *RPS6KA2* were found to be inherited in an autosomal-dominant pattern.

Several authors have combined WES and other strategies to identify potential candidate genes. WES identified alterations in *GREB1L*, a target of RA signalling, as responsible for Müllerian abnormalities, especially for MRKH Type II (Jacquinet et al., 2020). Müllerian ducts were absent in mouse models lacking Greb1l (De Tomasi et al., 2017). WES also found a rare homozygous variant in *CDC42BPB* in three sisters with septate uterus (Luo et al., 2020). Backhouse et al. (2019) used WES in combination with microarrays in patients with MRKH and found damaging variants of several genes: *LRP10*, encoding a lipoprotein receptor; *FRAS1*, encoding an extracellular matrix protein previously described as essential for metanephros organogenesis (Pitera et al., 2008); *RSPO4*, involved in the WNT pathway; *NPHP3*, which was previously linked to kidney development (Olbrich et al., 2003); *DYNC2H1*, a cilium-related dynein; and *NAALADL2*, which encodes an enzyme not yet linked to MDAs. This study also identified *MKKS*, the gene that triggers McKusick–Kaufman syndrome (OMIM 236700), presenting reproductive effects related to vaginal maldevelopment (Backhouse et al., 2019).

A separate WES study also found pathogenic mutations in the olfactory receptor genes *OR4M2* and *OR2T2*, the phosphodiesterase gene *PDE11A-AS1*, ribonucleoprotein-encoding *HNRNPCL1* and *ZNF816*, which encodes a zinc finger protein, in patients with MRKH Type I (Chen et al., 2015). These genes have not been directly linked to MDAs or Müllerian development. However, they could account for candidate causative genes to investigate further.

Model organisms are essential for the study of diseases, as demonstrated by efforts from the international community, such as the International Mouse Phenotyping Consortium (IMPC; www.mousephenotype.org) and Mouse Genome Informatics (MGI; http://www.informatics.jax.org) (Brown and Moore, 2012; Bult et al., 2019). The IMPC database aims for detailed and comprehensive high-throughput phenotyping of mouse knockout lines for every gene in the murine genome, whereas the MGI database catalogues the genetic alleles in murine model lines alongside genomic and biological information. Moreover, developmental disorders can specifically benefit from the creation of the Deciphering the Mechanisms of Developmental Disorders (DMDD; https://dmdd.org.uk/) database (Adams et al., 2013; Mohun et al., 2013), a project focused on mouse knockout lines displaying embryonic development disruption. These databases complement the investigation of the genetics of MDAs. Data mining of knockout phenotypes reporting aberrations of the Müllerian ducts is a promising strategy to discover new causative genes that could have previously been missed, shedding light on the origins and genetic contributions to MDAs (Moore et al., 2019). Furthermore, extensive phenotyping and transcriptional profiling would allow for study of gene expression, interactions, GRNs and pathways that are perturbed in MDAs.

## Conclusion

MDAs are a range of phenotypically heterogeneous disorders affecting the female reproductive system that originate from the aberrant development of the Müllerian duct. Despite the challenges that these malformations pose due to their complex multifactorial aetiology, the clinical picture they present and the *de novo* versus inherited nature of causative genetic variants, some advances have been made in the field. New insights into the biology underlying Müllerian pathology can be expected from studies on large cohorts of well-characterised patients in combination with state-of-the-art technologies such as NGS, transcriptomics, new model systems and genome-wide interrogation of comprehensive databases, opening potential therapeutic windows.

These approaches could greatly improve our understanding of the genomic aetiology of MDAs. This would highly benefit prevention as a key clinical strategy to address these disorders. Knowledge on the exact genetic/genomic MDAs cause would allow researchers to design a genetic testing panel to look at those variations in patients with Müllerian abnormalities, to achieve a better diagnosis. It would also improve clinical management. Furthermore, it would be possible to interrogate the genome of prospective parents and carry out preimplantation genetic diagnosis, as well as genetic screening in first-trimester pregnancy. Finally, understanding the link between causative genomic aberrations and MDAs would create the opportunity of offering genetic counselling to women coming from families with MDA cases to provide them with the right to an informed decision.

## Supplementary Material

Supplementary information
