# Supplementary material for: Studying Müllerian duct anomalies – from cataloguing phenotypes to discovering causation
Source: Dis Model Mech. 2021 Jun 23;14(6):dmm047977. doi: 10.1242/dmm.047977 (PMC8246269; doi:10.1242/dmm.047977)
Supplement: Supplementary information [file dmm-14-047977-s1.pdf]

Table S1. Genomic rearrangements in MDAs

Click here to download Table S1
